# COVID-19 and Aspiration Pneumonia: Similar Pulmonary Findings with Different Diagnoses—a Pitfall in [18F]FDG PET/CT

**DOI:** 10.1007/s42399-021-01030-y

**Published:** 2021-07-29

**Authors:** Virginia Liberini, Serena Grimaldi, Martin W. Huellner, Francesca Giunta, Costanza Bachi, Sara Dall’Armellina, Federica Onesti, Francesco Ceci, Carola Boccomini, Massimiliano Icardi, Désirée Deandreis

**Affiliations:** 1 Nuclear Medicine Unit, Department of Medical Sciences, University of Turin, Corso Dogliotti 14, 10126 Turin, Italy; 2 Department of Nuclear Medicine, University Hospital Zurich, Rämistrasse 100, CH-8091 Zurich, Switzerland; 3 Hematology Unit, Città della Salute e della Scienza University and Hospital, Turin, Italy; 4 2nd Medical Oncology Division, Città della Salute e della Scienza, Turin, Italy

**Keywords:** [18F]FDG PET/CT, COVID-19, Pneumonia, Ab ingestis, Aspiration pneumonia, SARS-CoV-2

## Abstract

Since December 2019, the severe acute respiratory syndrome coronavirus 2 (SARS-CoV-2) has become a worldwide pandemic. Especially in the centers most affected by the pandemic, symptoms (such as fever, cough, myalgia, or fatigue) and/or radiological signs (such as ground-glass opacity) typically related to COVID-19 often diverted clinicians’ attention from other diseases. Despite the urgency to recognize and cure SARS-CoV-2 infection, a plethora of differential diagnoses must be considered, and other diseases must be equally and promptly treated, as described in this case report.

## Introduction

The severe acute respiratory syndrome coronavirus 2 (SARS-CoV-2) has become a worldwide pandemic. The infective illness (COVID-19) may vary from asymptomatic disease to life-threatening pneumonia [1–3].

Computed tomography (CT) plays a key role in detecting COVID-19 pneumonia [4]. Moreover, few cases have been detected incidentally by ^18^F-fuorodeoxyglucose ([18F]FDG) positron emission tomography (PET)/CT [5–7].

Despite their high sensitivity, CT and PET/CT findings are unspecific and may overlap with other diseases [8] and may mislead from the right diagnosis, challenging the differential diagnosis and delaying the correct individual patient management for physicians.

We present two asymptomatic, immunocompromised [18F]FDG PET/CT patients from a hospital in a COVID-19 hotspot, having similar lung findings suspicious for COVID-19 pneumonia—one of which was false positive.

## Case Report

Patient 1 is an asymptomatic 80-year-old man with a history of non-Hodgkin lymphoma who underwent [18F]FDG PET/CT after the end of immunochemotherapy in March 2020.

**Fig. 1:**
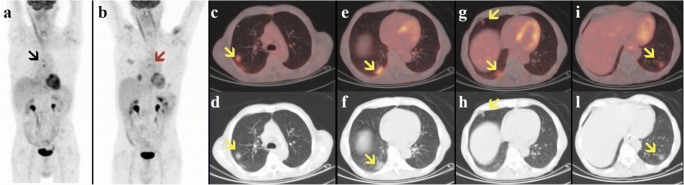
Previous (**a**) and current (**b–l**) [18F]FDG PET/CT of an asymptomatic 80-year-old man with a history of non-Hodgkin lymphoma. Maximum intensity projection (**a–b**), transaxial fused PET/CT (**c, e, g, i**), and CT (**d, f, h, l**) images of [18F]FDG uptake in the lungs

Compared with the previous PET/CT scan (Fig. 1a), the current PET/CT (Fig. 1b) showed resolution of mediastinal lymph node uptake (black arrow). However, PET/CT and CT images (Fig. 1c–l) revealed the appearance of multiple bilateral FDG-avid ground-glass opacities (GGOs, yellow arrows), with a predominantly peripheral distribution in the posterior segments of the inferior lung lobes. Moreover, new mildly increased uptake was seen in several mediastinal lymph nodes, without enlargement on CT images (Fig. 1b, red arrow).

Ground-glass opacities are the most common CT finding in COVID-19 pneumonia, particularly in the early phase of the disease, especially in asymptomatic patients [9]. The pattern is usually multifocal, bilateral, and peripheral, with a posterior distribution, mainly in the lower lobes, while enlarged mediastinal or hilar lymph nodes are not typically observed [4, 10, 11].

FDG uptake has been described in COVID-19 pneumonia–related GGOs in both symptomatic and asymptomatic patients [5, 6, 12, 13], as well as in mediastinal lymph nodes [14].

Due to these findings, the patient was counseled, and isolation procedures and scanner sanitation measures were started. The gold standard for the diagnosis of COVID-19 infection, a reverse transcriptase-polymerase chain reaction (RT-PCR) test from pharyngeal swabs, was subsequently performed and confirmed the diagnosis of COVID-19 [1, 15].

Patient 2 is an asymptomatic 51-year-old man with a history of oral cavity squamous cell carcinoma, associated swallowing difficulties, and repetitive aspiration pneumonia, previously treated by surgery, chemotherapy, and immunotherapy, who underwent [18F]FDG PET/CT for restaging in March 2020.

**Fig. 2:**
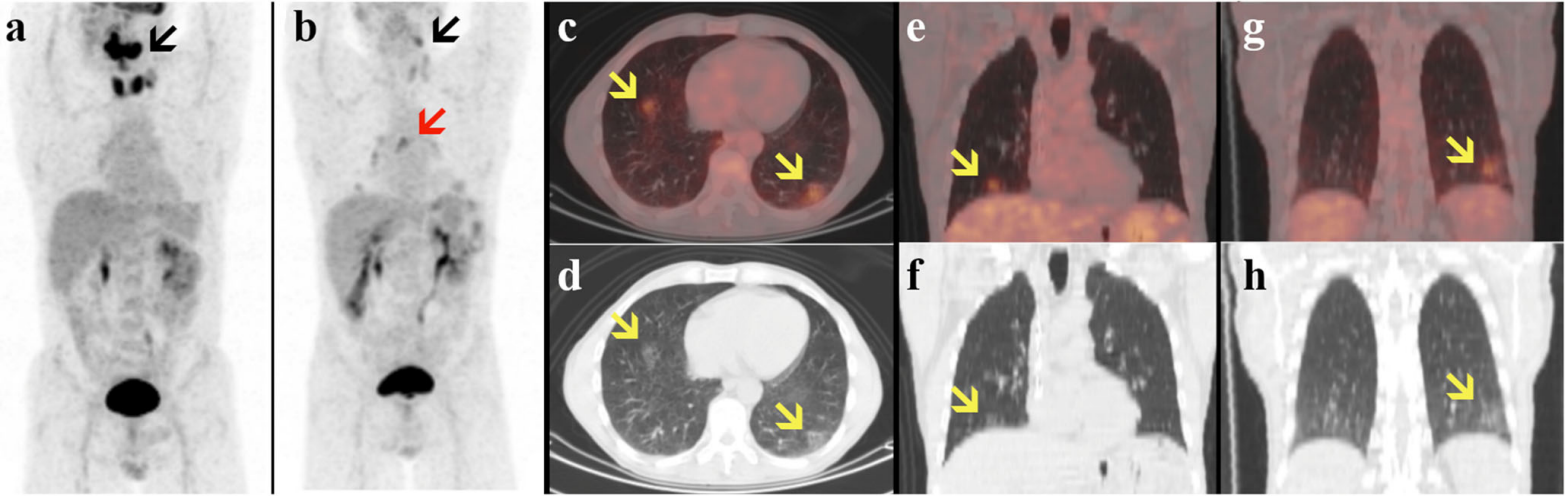
Previous (**a**) and current (**b–l**) [18F]FDG PET/CT of an asymptomatic 51-year-old man with a history of oral cavity squamous cell carcinoma. Maximum intensity projection (**a–b**), transaxial and coronal fused PET/CT (**c, e, g**), and CT (**d, f, h**) images of [18F]FDG uptake in the lungs

Follow-up PET/CT (Fig. 2b) showed a good partial metabolic response of the primary tumor (black arrows) compared with the baseline scan (Fig. 2a). However, PET/CT showed the appearance of few bilateral GGOs (yellow arrows) in the posterior segments of the inferior lung lobes (Fig. 2 c–h) associated with hilar and mediastinal lymph nodes (Fig. 2b, red arrow), both mildly FDG-avid.

Due to these findings suspicious for COVID-19, the patient was counseled, and isolation and sanitation measures were triggered. The patient was quarantined at home with adequate clinical monitoring. After 2 days, he developed fever > 38°C, which resolved spontaneously. RT-PCR for SARS-CoV-2 was negative in two consecutive tests.

In this patient, considering the clinical history, [18F]FDG PET/CT findings matched aspiration pneumonia, mimicking COVID-19 infection [16, 17].

## Discussion

The SARS-CoV-2 pandemic has a considerable impact on nuclear medicine departments worldwide, and also false-positive cases may entail enormous efforts. While several articles have reported and suggested potential clinical usefulness of [18F]FDG PET/CT in patients with suspected COVID-19 infection, especially at early stages when clinical symptoms are nonspecific, only Treglia recently emphasized the risk of considering these findings as a “peculiar metabolic behavior of this infection” [18].

Different conditions, both infectious and noninfectious, are responsible for bilateral lung parenchymal involvement, developing either as focal or diffuse lung disease. Most of these diseases are characterized by an increased tropism for glucose by inflammatory cells recalled and activated by the infectious process or by tumor cells, translating into an increased uptake of [18F]FDG related to the increased glucose metabolism. PET/CT with [18F]FDG helps the characterization of these pathological processes; however, some of these, especially in the early stages of disease, may be difficult to distinguish from each other. Several studies have highlighted the few differences present radiologically and metabolically between COVID-19 infection and other viral pneumonias: COVID-19 pneumonia is more likely to have a peripheral distribution (80% vs. 57%), ground-glass opacity (91% vs. 68%), and fine reticular opacity (56% vs. 22%), but less likely to have a central plus peripheral distribution (14% vs. 35%) and pleural effusion (4% vs. 39%) [19–21].

However, to the best of our knowledge, there is currently no evidence in the literature regarding the radiological and metabolic differences between COVID-19 and aspiration pneumonia. Aspiration pneumonia presents on [18F]FDG PET/CT with different patterns, usually very striking, as this clinical condition often leads to severe over-infection, with the development of lobar or segmental pneumonia, bronchopneumonia, lung abscess, and empyema. Frequently, PET/CT shows increased diffuse, intense, and bilateral uptake especially in the posterior segment of the upper lobes and the superior segment of the lower lobes, which may mimic other FDG-avid pulmonary diseases. Nevertheless, in some cases, especially if diagnosed early, aspiration pneumonia may occur only with unilateral or bilateral ground glass, especially in cases of diffuse aspiration bronchiolitis in patients with esophageal conditions such as achalasia, Zenker’s diverticulum, or carcinoma of the oral cavity, especially esophageal, associated with dysphagia, regurgitation, and aspiration [22].

Therefore, there is currently no scientific evidence about the usefulness of [18F]FDG PET/CT in the evaluation of COVID-19 pneumonia, as metabolic behavior of this infection is not pathognomonic, but rather nonspecific. On the other hand, one should be more careful about the not remote possibility of identifying with [18F]FDG PET/CT an interstitial pneumonia (with ground-glass opacity) suspected of COVID-19 infection [18].

To the best of our knowledge, this report is the first to present a concrete example of the lack of specificity of PET/CT findings in COVID-19 pneumonia comparing to aspiration pneumonia. In case of PET/CT findings suggestive for COVID-19 pneumonia, asymptomatic patients should be carefully monitored or tested with RT-PCR, but at the same time, the differential diagnosis must be considered.

This case highlights how differential diagnosis on imaging remains a challenge, with the additional reason of providing proper individual patient management and avoiding overload of the COVID-19 surveillance system.

## Data Availability

Not applicable. Code Availability Not applicable.
